# The Role of Sirt1 in Bile Acid Regulation during Calorie Restriction in Mice

**DOI:** 10.1371/journal.pone.0138307

**Published:** 2015-09-15

**Authors:** Zidong Donna Fu, Julia Yue Cui, Curtis D. Klaassen

**Affiliations:** 1 Department of Pharmacology, Toxicology, and Therapeutics, University of Kansas Medical Center, Kansas City, KS, United States of America; 2 Department of Pharmacology, Harbin Medical University (the State-Province Key Laboratories of Biomedicine-Pharmaceutics of China), Harbin, Heilongjiang Province, People's Republic of China, 150081; 3 Department of Environmental and Occupational Health Sciences, University of Washington, Seattle, WA, United States of America; 4 Department of Internal Medicine, University of Kansas Medical Center, Kansas City, KS, United States of America; CIMA. University of Navarra, SPAIN

## Abstract

Sirtuin 1 (Sirt1) is an NAD^+^-dependent protein deacetylase that is proposed to mediate many health-promoting effects of calorie restriction (CR). We recently reported that short-term CR increased the bile acid (BA) pool size in mice, likely due to increased BA synthesis in liver. Given the important role of Sirt1 in the regulation of glucose, lipid, as well as BA metabolism, we hypothesized that the CR-induced increase in BAs is Sirt1-dependent. To address this, the present study utilized genetically-modified mice that were Sirt1 loss of function (liver knockout, LKO) or Sirt1 gain of function (whole body-transgenic, TG). Three genotypes of mice (Sirt1-LKO, wild-type, and Sirt1-TG) were each randomly divided into *ad libitum* or 40% CR feeding for one month. BAs were extracted from various compartments of the enterohepatic circulation, followed by BA profiling by UPLC-MS/MS. CR increased the BA pool size and total BAs in serum, gallbladder, and small intestine. The CR-induced increase in BA pool size correlated with the tendency of increase in the expression of the rate-limiting BA-synthetic enzyme Cyp7a1. However, in contrast to the hypothesis, the CR-induced increase in BA pool size and Cyp7a1 expression was still observed with ablated expression of Sirt1 in liver, and completely suppressed with whole-body overexpression of Sirt1. Furthermore, in terms of BA composition, CR increased the ratio of 12α-hydroxylated BAs regardless of Sirt1 genotypes. In conclusion, the CR-induced alterations in BA pool size, BA profiles, and expression of BA-related genes do not appear to be dependent on Sirt1.

## Introduction

The sirtuins are an evolutionarily conserved family of proteins that regulates various important physiological processes, such as aging, metabolic homeostasis, inflammation, as well as cancer. Among the seven sirtuins in mammals, sirtuin 1 (Sirt1) has the highest orthology to the well-known longevity-regulator Sir2 in lower organisms [1]. Extensive evidence using natural [2,3] or synthetic small molecule activators [4,5] or genetically-modified mice [6] have demonstrated that Sirt1 plays a role in the delayed onset of aging-related diseases.

Calorie restriction (CR), which is reduced calorie intake without causing malnutrition, delays the onset of various aging-related diseases, such as metabolic disorders, cardiovascular and neurological dysfunction, as well as cancer in various species [7]. CR increases the expression of Sirt1 in various tissues, including muscle, brain, heart, liver, kidney, pancreas, and adipose tissue [8,9,10,11]. Sirt1 is considered to mediate some of the health-promoting effects of CR [12,13]. Resveratrol partially mimics the beneficial health effects of CR by activating Sirt1 [14,15].

Sirt1 functions as a key metabolic sensor and mediates homeostatic responses to nutrient availability. Sirt1 overexpression or activation reduces fat gain and improves insulin sensitivity in mice [4,16,17]. Small molecule activators of human SIRT1 have become therapeutic candidates that are currently in clinical trials for the treatment of type 2 diabetes [5]. It was revealed more recently that NAD+ boosters could stimulate Sirt1 and improves metabolic health [18,19]. Sirt1 is a master regulator of transcriptional networks that control hepatic metabolism of fatty acids and cholesterol [20]. Sirt1 protects against pathologies induced by a high-fat diet, such as glucose intolerance, liver steatosis and inflammation [21,22,23,24]. Furthermore, Sirt1 promotes fat mobilization and stimulates the conversion of white adipose to brown adipose tissue [25].

Bile acids (BAs), the endogenous metabolic end-product of cholesterol in liver, have recently been shown to be important signaling molecules and metabolic regulators that control glucose and lipid homeostasis as well as energy consumption [26,27]. The rate-limiting synthetic enzyme, cholesterol 7α-hydroxylase (Cyp7a1) controls the overall rate of BA production [28] and determines the BA pool size [29]. After synthesis in liver and storage in gallbladder, BAs are secreted into the intestinal lumen, where secondary BAs are formed by intestinal bacteria. The majority of BAs are reabsorbed from the end of the small intestine and return to the liver through the portal blood. This cycle is called the enterohepatic circulation, and it is promoted by multiple BA transporters in both liver and intestine. The BA receptor farnesoid X receptor (FXR) plays a key role in controlling BA homeostasis [30].

In addition to regulating glucose and lipid metabolism, recent studies have revealed important roles of Sirt1 in bile acid metabolism and transport in liver and intestine [31,32]. Our previous report showed that short-term CR could increase the BA pool size in mice [33]. Given the important role of Sirt1 in metabolic homeostasis, we hypothesized that CR-induced changes of BA metabolism are dependent on Sirt1. Thus, Sirt1 genetically-modified mice were utilized, including Sirt1-liver knockout (loss of function) and Sirt1-whole body-transgenic (gain of function) mice, to investigate the role of Sirt1 in BA regulation during CR. A highly sensitive and accurate analytical method, ultra-performance liquid chromatography-tandem mass spectrometry (UPLC-MS/MS), was applied to determine both BA concentrations and composition in various specimens to reveal the enterohepatic metabolism and circulation of BAs. Moreover, the expression of BA-related genes were quantified to provide mechanistic explanation to the CR-induced changes in BA concentrations and composition.

## Materials and Methods

### Ethics Statement

This study was carried out in strict accordance with the recommendations in the Guide for the Care and Use of Laboratory Animals of the National Institutes of Health. This study was approved by the Institutional Animal Care and Use Committee at the University of Kansas Medical Center.

### Animal Experiments and Study Design

Sirt1-LKO mice with hepatocyte-specific ablation of Sirt1 were bred by crossing Sirt1-flox/flox mice and Albumin-Cre mice, which were kindly provided by Dr. Xiaoling Li (National Institutes of Health, Research Triangle Park, US) [22]. Sirt1-LKO mice have ablated expression of Sirt1 only in liver and age-matched Lox littermates (Albumin-Cre negative, Sirt1-flox/flox) were used as controls. Whole-body Sirt1-TG mice, with the murine Sirt1 gene cloned into a BAC vector, were obtained from Dr. Manuel Serrano (Spanish National Cancer Research Center, Madrid, Spain) [24]. Sirt1-transgenic mice have over-expression of Sirt1 in all tissues, and wild-type (WT) littermates were used as controls. The present study utilized only male mice (8 weeks of age, in C57BL/6 background). All mice were housed in an AAALAC-accredited facility at the University of Kansas Medical Center, with a 14-h light/10-h dark-cycle, temperature- and humidity-controlled environment and *ad libitum* (AL) access to water. Because Lox controls and WT mice were similar in terms of BAs, only WT controls were shown to simplify data presentation.

Each genotype of mice were randomly divided into AL and 40% CR feeding groups (n = 5). All mice were housed individually and the AL-fed mice were given AL access to purified AIN-93M diet (TD94048, Harlan Teklad, Madison, WI) throughout this study. The diet consumption of the AL group was recorded daily, and subsequently 60% of this amount of CR-diet (TD110468, Harlan Teklad, Madison, WI) were given to the 40% CR group for one month. Feed was provided between 5–6 PM daily. CR mice finished their daily feed within 2 h after feeding. Feed to CR groups were gradually decreased one week before start of the one-month CR feeding, to prevent sudden weight loss as previously reported [33]. Custom enriched diets were given to CR groups to prevent malnutrition. The AL diet TD94048 contained 12.4% protein, 68.3% carbohydrate, and 4.1% fat. The CR diet TD110468 contained 20.6% protein, 54.1% carbohydrate, and 6.9% fat.

### Sample Collection

AL-fed mice were given full access to food until tissue collection, and CR-fed mice finished their daily allotment of food by 8 PM the day before tissue collection. Mice were anesthetized with pentobarbital (50 mg/kg), body weights were recorded and blood collected by retro-orbital bleeding. Blood was kept on ice and centrifuged at 4,000 rpm at 4°C for 20 min. Serum was separated and stored at -80°C. Gallbladder (GB) tissue was removed intact without disrupting the bile inside, weighed, and snap-frozen in liquid nitrogen. The liver was removed, weighed, and snap-frozen. The contents of the small (SI) and large intestine (LI) were collected separately by flushing into tubes filled with 10 mL of ice-cold saline. The SI tissue was separated into duodenum, jejunum, and ileum, and snap-frozen. All samples were stored at -80°C. Tissue collections were between 9 AM and noon.

### BA Extraction and Quantification

BAs were extracted from serum, liver, gallbladder, and intestine [33] and quantified by UPLC-MS/MS as previously described [34,35]. BAs quantified included CA (cholic acid), CDCA (chenodeoxycholic acid), αMCA (muricholic acid), βMCA, DCA (deoxycholic acid), LCA (lithocholic acid), UDCA (ursodeoxycholic acid), MDCA (murideoxycholic acid), ωMCA, and HDCA (hyodeoxycholic acid) as well as their taurine (T) conjugates. The concentrations of individual BAs were summed to derive the concentrations of total BAs.

### Multiplex Suspension Assay

Total RNA was isolated from liver and ileum tissues using RNA Bee reagent (Tel-Test Inc., Friendswood, TX). The mRNAs of genes encoding BA synthetic enzymes (Cytochrome P450s Cyp7a1, Cyp8b1, Cyp27a1, and Cyp7b1), BA transporters in liver [Na+/taurocholate cotransporting polypeptide (Ntcp), organic anion transporting polypeptide 1b2 (Oatp1b2), and bile salt export pump (Bsep)] and ileum [apical sodium-dependent bile acid transporter (Asbt) and organic solute transporters (Ostα/Ostβ)] were quantified by Panomics 2.0 QuantiGene Plex technology (Panomics/Affymetrix Inc., Fremont, CA). Probe sets for individual genes were designed by Panomics/Affymetrix Inc. with Panel numbers 21150 and 21383 (http://www.panomics.com). Fluorescence was analyzed using a Bio-Plex 200 system array reader with Luminex 100 X-MAP technology, and data were acquired using Bio-Plex data manager software 5.0 (Bio-Rad, Hercules, CA). The mRNAs of target genes were normalized to Gapdh.

### Reverse Transcription and Quantitative Real-time PCR Analysis

Total RNA was transcribed to single-stranded cDNA using a High Capacity cDNA Reverse Transcription Kit 1001073 (Applied Biosystems, Foster City, CA), and the cDNA products were amplified by PCR, using Power SYBR Green PCR Master Mix in a 7900HT Fast Real-Time PCR System (Applied Biosystems, Foster City, CA). The mRNAs of genes encoding BA conjugating enzymes [bile acid-CoA ligase (BAL) and bile acid-CoA: amino acid *N*-acyltransferase (BAT)], ileal BA binding protein (Ibabp), and proteins involved in Cyp7a1 regulation [farnesoid X receptor (FXR), small heterodimer partner (SHP), liver receptor homolog-1 (LRH-1), hepatocyte nuclear factor 4 alpha (HNF4α), fibroblast growth factor 15 (Fgf15), fibroblast growth factor receptor 4(Fgfr4), and peroxisome proliferator-activated receptor gamma coactivator 1-alpha (PGC-1α)], as well as Sirt1 were quantified and normalized to β-actin. The sequences of real-time PCR primers (Integrated DNA Technologies, Coralville, IA) were reported previously [33].

### Statistical Analysis

Data are presented as mean ± SEM. To compare Sirt1-LKO, WT, and TG mice, data were analyzed by one-way ANOVA, followed by Duncan’s post-hoc test, differences being considered significant at *p* < 0.05 (#). To compare AL- and CR-fed mice, data were analyzed by Student's *t*-test, differences being considered significant at *p* < 0.05 (*).

## Results

### Body Weight, Liver Weight, and Sirt1 Expression

**Fig 1:**
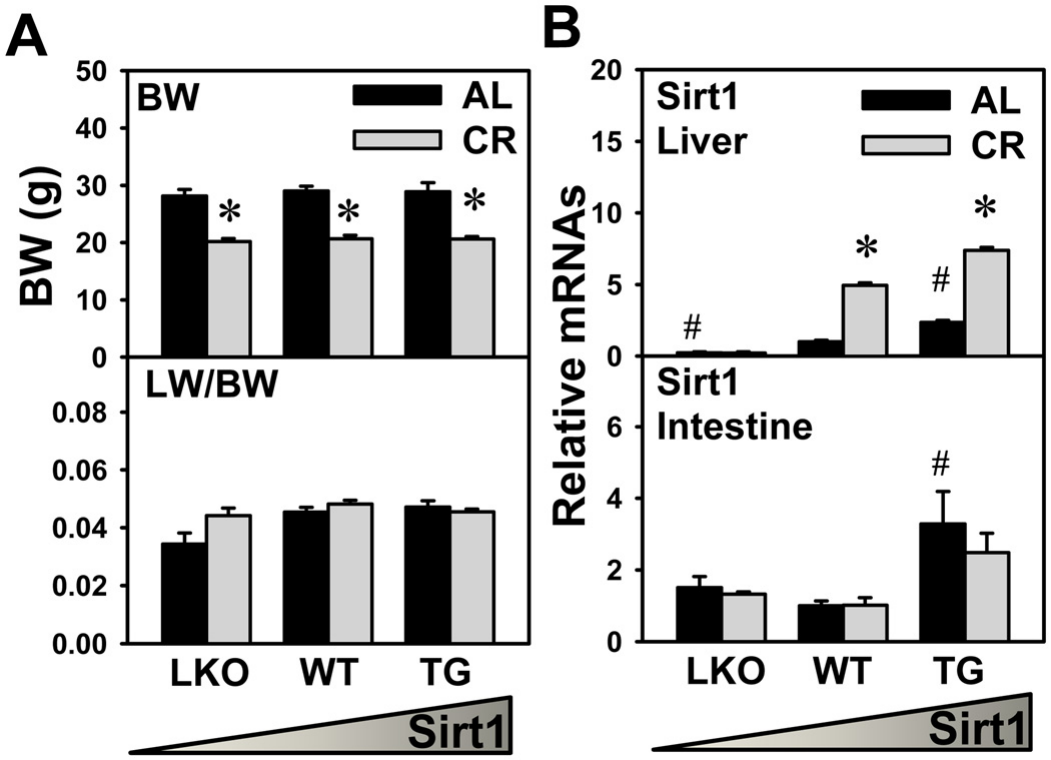
Body weight, liver weight, and Sirt1 expression. After one-month of 40% CR, body weight (BW) and liver weight (LW) of Sirt1-LKO, WT, and Sirt1-TG mice were recorded. (A) The ratio of LW to BW was calculated. (B) The expression of Sirt1 was quantified in liver and ileum of three genotypes of mice that were either AL- or CR-fed. Data are presented as means ± SEM of 5 mice. The triangle represents increased expression of Sirt1 in liver. # represents differences compared to WT mice, by one-way ANOVA, followed by Duncan’s post-hoc test (*p* < 0.05). * represents differences compared to AL mice of the same genotype by Student's *t*-test (*p* < 0.05).

Body weight and liver weight were recorded in all genotypes of mice either AL- or CR-fed. After one-month of 40% CR, body weights of CR mice were all approximately 28% lower than AL mice in all three genotypes of Sirt1 mice (Fig 1A). The ratio of liver weight and body weight remained relatively constant during CR, regardless of Sirt1 genotypes.

The expression of Sirt1 in both liver and intestine were analyzed by real-time PCR. As shown in Fig 1B, Sirt1 expression in liver was ablated in Sirt1-LKO mice, and more abundant in Sirt1-TG mice (2.3-fold) than WT mice. CR did not induce the hepatic Sirt1 expression in Sirt1-LKO mice. CR induced the hepatic expression of Sirt1 in both WT and Sirt1-TG mice (3.9-fold and 2.1-fold, respectively). In ileum, Sirt1 expression in Sirt1-LKO mice was similar to WT mice, and Sirt1-TG mice had over-expression of Sirt1 (2.3-fold) compared to WT mice. In addition, Sirt1 expression in ileum remained relatively constant after CR in all genotypes of mice.

### The BA Pool Size and Total BAs in Various Compartments of Enterohepatic Circulation

**Fig 2:**
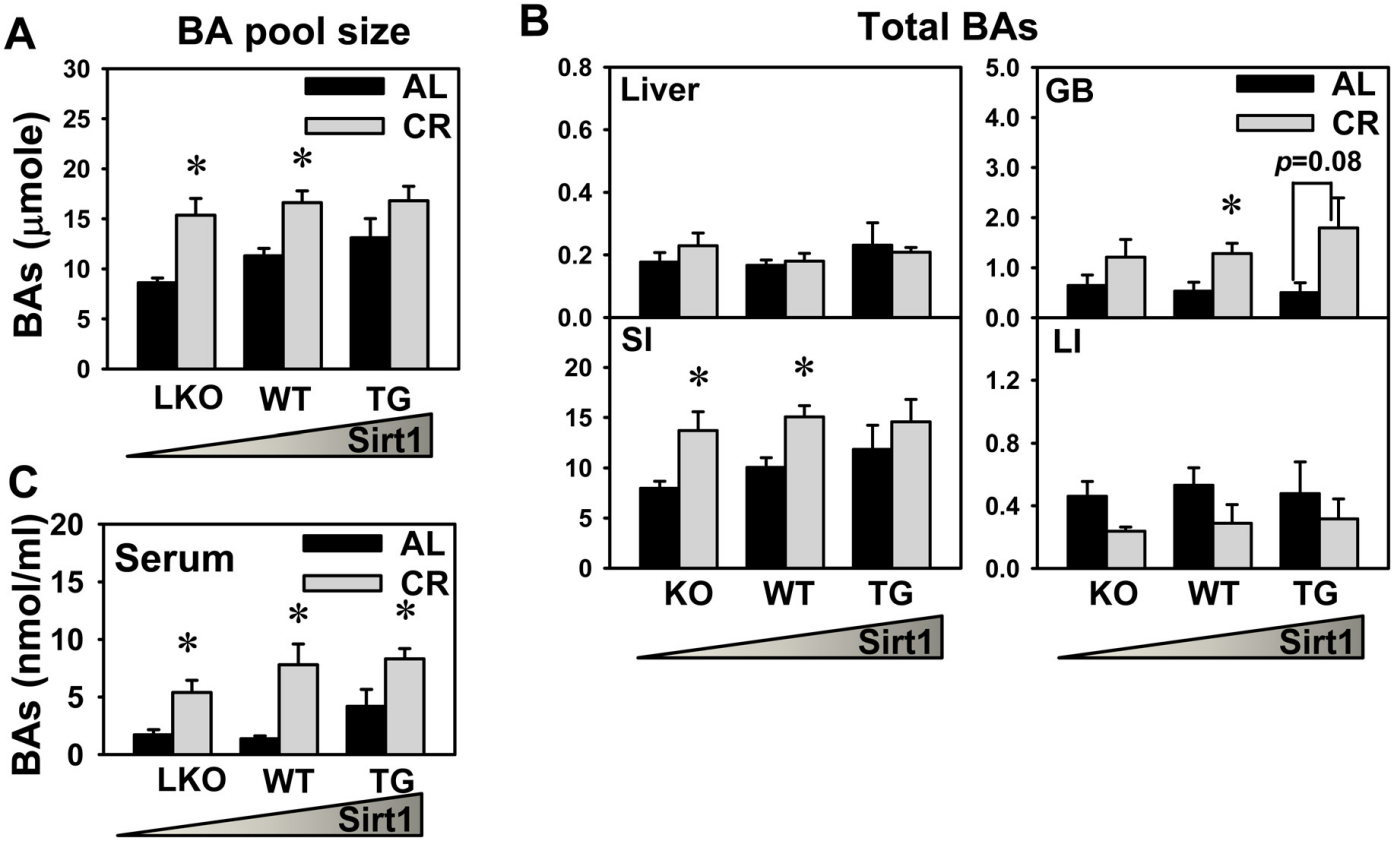
The BA pool size and total BAs in various compartments of enterohepatic circulation. Sirt1-LKO, WT, and Sirt1-TG mice were given ad libitum (AL) or 40% CR feeding (n = 5) for one month. BAs were extracted from liver, gallbladder (GB), small (SI) and large intestine (LI), and twenty major individual BAs were quantified by UPLC-MS/MS. The BA pool size (A) is estimated by adding total BAs in liver, GB, and intestine (B). (C) shows total BAs in serum. Data are presented as means ± SEM of 5 mice. The triangle represents increased expression of Sirt1 in liver. # represents differences compared to WT mice, by one-way ANOVA, followed by Duncan’s post-hoc test (*p* < 0.05). * represents differences compared to AL mice of the same genotype by Student's *t*-test (*p* < 0.05).

BA pool size is defined as the total amount of BAs circulating in the enterohepatic circulation [28]. BAs were quantified by UPLC-MS/MS in various compartments of the enterohepatic circulation. BA pool size was estimated by adding total BAs in liver, gallbladder (GB), and intestine, as previously reported [30,33]. As shown in Fig 2A, the BA pool size under AL feeding tended to increase with increased Sirt1 expression, but it was not statistically significant. CR increased the BA pool size in WT as well as Sirt1-LKO mice, but did not cause a statistically significant change in the BA pool size in Sirt1-TG mice. Under AL feeding, total BA concentrations in liver, GB, SI, and LI were similar in Sirt1-LKO, WT, and Sirt1-TG mice (Fig 2B). CR did not cause a statistically significant change in total BAs in livers of Sirt1-LKO, WT, or Sirt1-TG mice. CR did not cause a statistically significant change in total BAs in GBs of Sirt1-LKO mice, but increased total BAs in GBs of WT mice and tended to increase total BAs in GBs of Sirt1-TG mice. CR increased total BAs in the SI of WT as well as the Sirt1-LKO mice, but did not cause a statistically significant change in total BAs in the SI of Sirt1-TG mice. CR did not cause a statistically significant change in total BAs in the LI of Sirt1-LKO, WT, or Sirt1-TG mice. Changes in total BAs in the SI were similar to those of the BA pool size, because BAs in the SI contents contribute over 85% of the BA pool.

The amount of BAs in serum is several orders of magnitude less than that in liver, GB, or intestine, so serum data are not required to determine BA pool size. BA concentrations in serum are important for systemic functions, such as the regulation of energy metabolism in muscle and adipose tissue. Therefore, BA concentrations in serum was quantified in the present study as well. It was found that CR markedly increased BA concentrations in serum in Sirt1-LKO, WT, as well as Sirt1-TG mice (Fig 2C).

### Concentrations of 12α-Hydroxylated BAs in GB and SI

**Fig 3:**
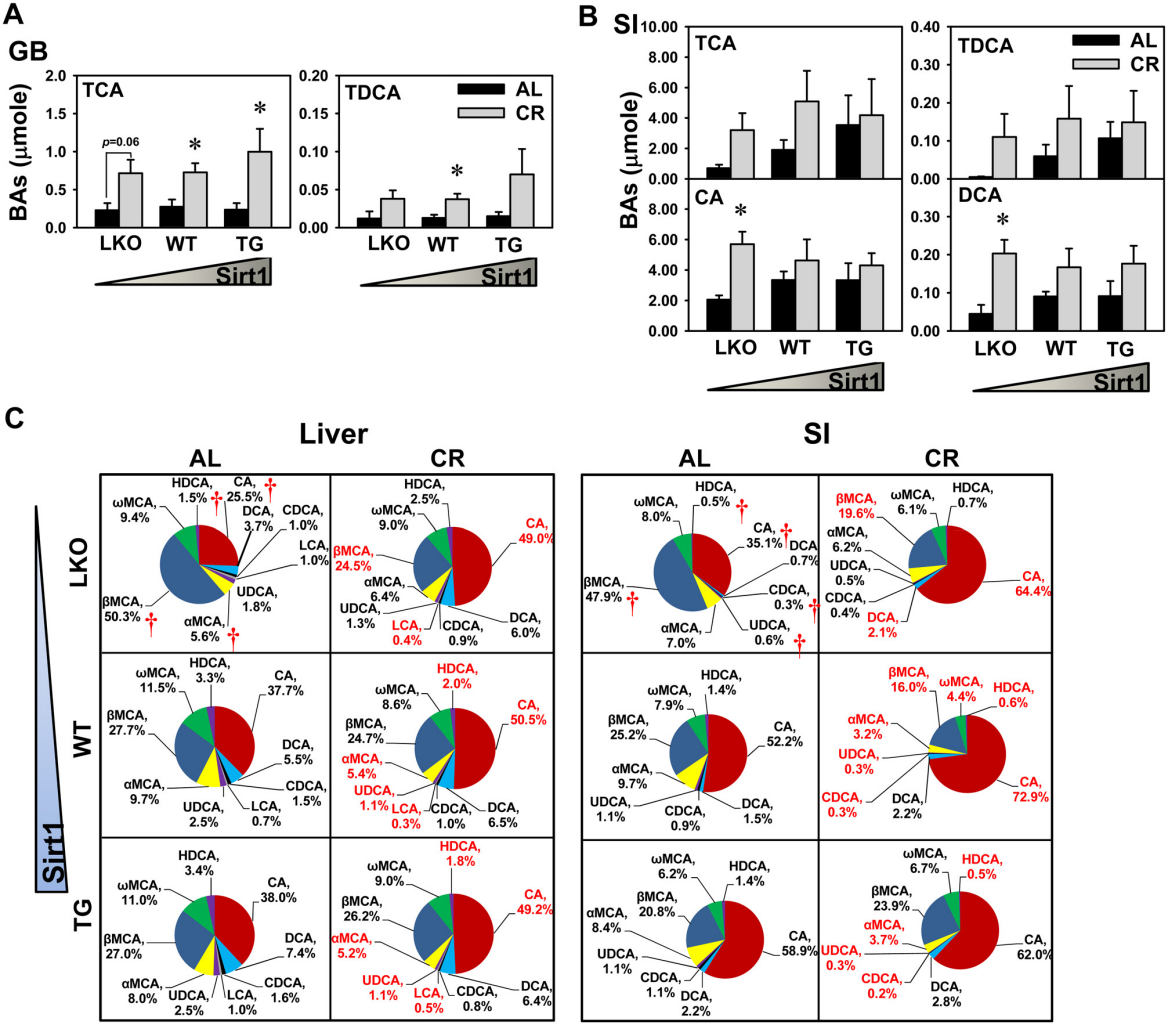
Concentrations of 12α-hydroxylated BAs in GB and SI and alteration of BA composition in liver and SI. CA and DCA as well as their taurine-conjugates have –OH group at 12α-C position, and therefore are referred to as 12α-hydroxylated BAs. (A) The concentrations of TCA and TDCA in GB and (B) (T)CA and (T)DCA in SI were shown. Data are presented as means ± SEM of 5 mice. The triangle represents increased expression of Sirt1 in liver. * represents differences compared to AL mice of the same genotype by Student's *t*-test (*p* < 0.05). (C) The alteration of BA composition in liver and SI were presented as means of 5 mice. For instance, the red portion of the pie chart represents the proportion of CA and TCA in total BA concentrations. † represents differences compared to WT mice, by one-way ANOVA, followed by Duncan’s post-hoc test (*p* < 0.05). BA and it percentage in red represent differences compared to AL mice of the same genotype by Student's *t*-test (*p* < 0.05).

CA and DCA are 12α-hydroxylated and therefore are referred to as 12α-hydroxylated BAs. It was reported that low levels of 12α-hydroxylated BAs appear to link hepatic insulin signaling in type 2 diabetes with dyslipidemia [36]. As shown in Fig 3A, CR increased TCA concentration in GBs of WT and Sirt1-TG mice, and tended to increase TCA concentration in GBs of Sirt1-LKO mice. CR increased TDCA concentration in GBs of WT mice, but did not alter TDCA concentration in GBs of Sirt1-LKO or Sirt1-TG mice. CR did not significantly alter TCA or TDCA in the SI of any of the three genotypes of mice (Fig 3B). CR increased CA and DCA concentrations in SI of Sirt1-LKO mice, but did not alter CA or DCA concentrations in SI of WT or Sirt1-TG mice.

### Alteration of BA Composition in Liver and SI

Individual BAs have different detergent properties to promote hepatic elimination of cholesterol and intestinal absorption of dietary lipids [37], as well as potencies to activate FXR [38,39,40] and TGR5 [41] receptors to regulate BAs, nutrients, and energy homeostasis. Therefore, in addition to BA concentrations, BA composition is another important aspect to examine in BA homeostasis. BA composition was analyzed in liver and SI of various genotypes of mice that were either AL- or CR-fed. As shown in Fig 3C, under AL feeding, the proportions of CA, αMCA, and HDCA in livers of Sirt1-LKO mice were lower than those in WT mice. CR increased the proportion of CA and decreased the proportions of LCA, UDCA, αMCA, and HDCA in livers of WT mice. Similarly, CR increased the proportion of CA and decreased proportions of LCA and βMCA in livers of Sirt1-LKO mice, and CR increased the proportion of CA and decreased the proportions of LCA, UDCA, αMCA, and HDCA in livers of Sirt1-TG mice. Under AL feeding, the proportions of CA, CDCA, UDCA, and HDCA in the SI of Sirt1-LKO mice were lower than those in WT mice, whereas the proportion of βMCA was higher. CR increased the proportion of CA and decreased the proportions of CDCA, UDCA, αMCA, βMCA, ωMCA, and HDCA in the SI of WT mice. Similarly, CR increased the proportions of CA and DCA and decreased the proportion of βMCA in SI of Sirt-1 LKO mice, and CR decreased the proportions of CDCA, UDCA, αMCA, and HDCA in SI of Sirt1-TG mice.

### Expression of BA-Synthetic and Conjugating Enzymes in Liver

**Fig 4:**
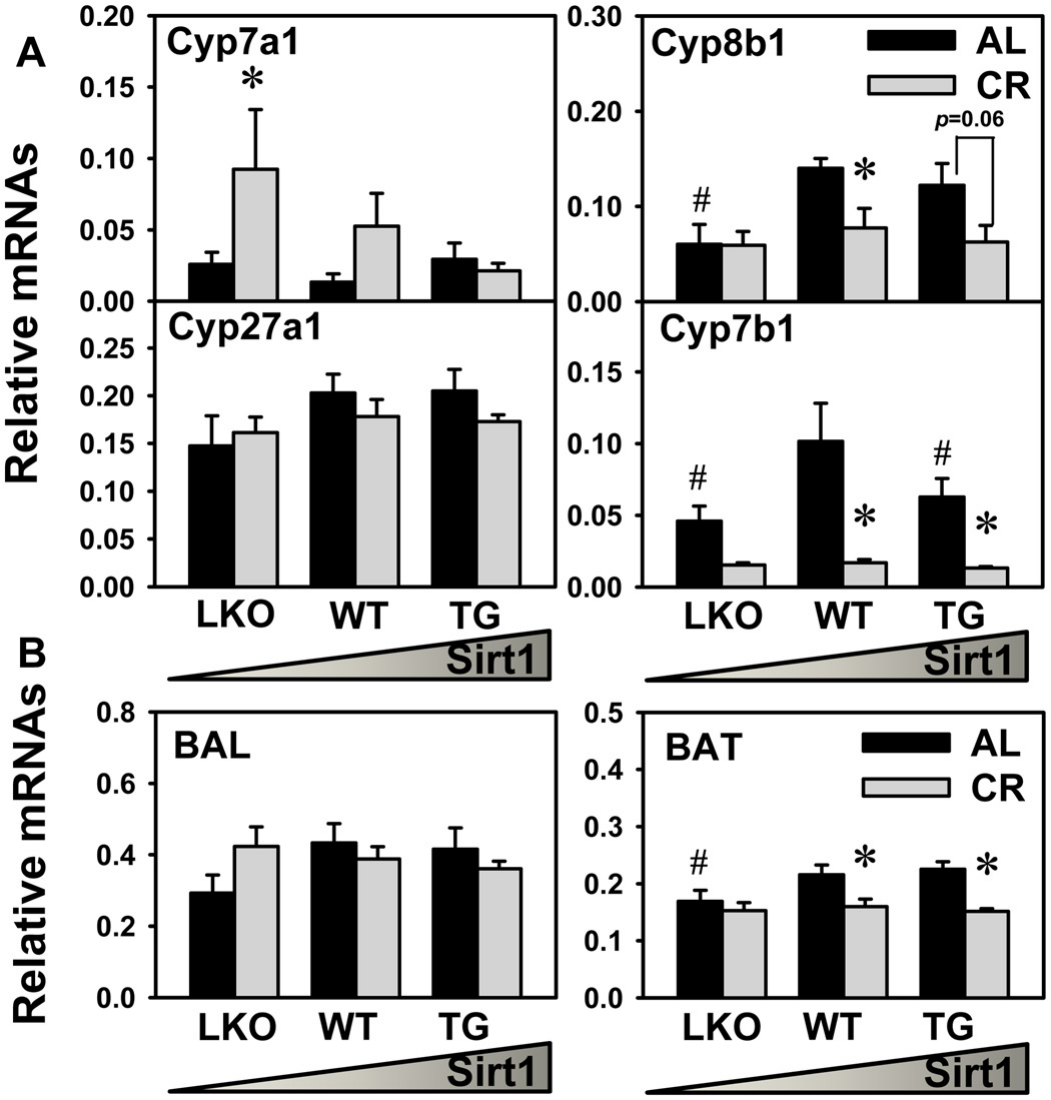
Expression of BA-synthetic and conjugating enzymes in liver. Total RNA was extracted from livers of Sirt1-LKO, WT, and Sirt1-TG mice that were given either AL or CR feeding. The mRNAs of (A) BA-synthetic (Cyp7a1, Cyp8b1, Cyp27a1, and Cyp7b1) and (B) BA-conjugating enzymes (BAL and BAT) were quantified. Data are presented as means ± SEM of 5 mice. The triangle represents increased expression of Sirt1 in liver. # represents differences compared to WT mice, by one-way ANOVA, followed by Duncan’s post-hoc test (*p* < 0.05). * represents differences compared to AL mice of the same genotype by Student's *t*-test (*p* < 0.05).

In order to provide insights on the mechanism for BA changes by CR in various genotypes of mice, expression of genes related to BA metabolism and transport were analyzed. Fig 4A showed the expression of BA synthetic enzymes (Cyp7a1, 8b1, 27a1, and 7b1) in liver. CR tended to increase Cyp7a1 mRNA (2.9-fold) in WT mice, increased Cyp7a1 mRNA (2.6-fold) in Sirt1-LKO mice, but did not alter Cyp7a1 mRNA in Sirt1-TG mice. Under AL feeding, Cyp8b1 mRNA in Sirt1-LKO mice was less than half of that in WT mice. CR decreased Cyp8b1 mRNA in half in WT and Sirt1-TG mice, but did not alter Cyp8b1 mRNA in Sirt1-LKO mice. Cyp27a1 mRNA was similar in Sirt1-LKO, WT, and Sirt1-TG mice under AL feeding, and CR did not alter Cyp27a1 mRNA in these mice. CR decreased Cyp7b1 mRNAs in Sirt1-LKO (67%), WT (83%), and Sirt1-TG (79%) mice. Fig 4B shows the expression of BA-conjugating enzymes in liver. BAL mRNA was similar in Sirt1-LKO, WT, and Sirt1-TG mice under AL feeding, and CR did not alter BAL mRNA in these mice. CR decreased BAT mRNAs in WT and Sirt1-TG mice, but did not alter BAT mRNA in Sirt1-LKO mice.

### Cyp7a1 Transcription Regulators in Liver and Ileum

**Fig 5:**
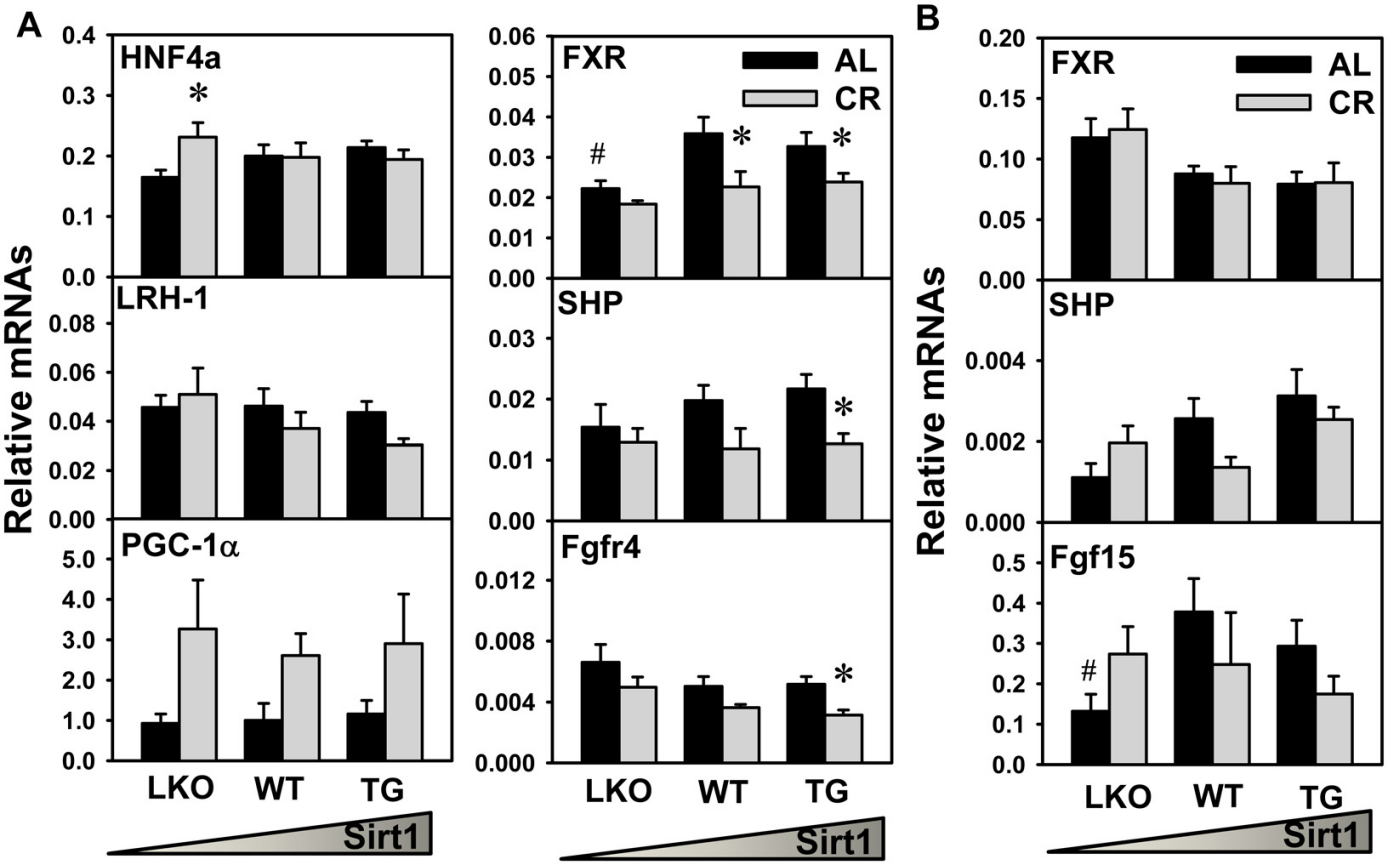
Expression of Cyp7a1 transcription regulators in liver and ileum. The mRNAs of key factors that regulate basal Cyp7a1 transcription (HNF4α, LRH-1, and PGC-1α) and BA-feedback inhibition of Cyp7a1 transcription (both FXR-SHP in liver and FXR-Fgf15 in ileum) were quantified in (A) liver and (B) intestine. Data are presented as means ± SEM of 5 mice. The triangle represents increased expression of Sirt1 in liver. # represents differences compared to WT mice, by one-way ANOVA, followed by Duncan’s post-hoc test (*p* < 0.05). * represents differences compared to AL mice of the same genotype by Student's *t*-test (*p* < 0.05).

In order to provide explanation for the CR-induced alterations of Cyp7a1 expression, the expression of multiple Cyp7a1 transcription regulators (positive: HNF4α, LRH-1, and PGC-1α; negative: FXR, SHP, Fgf15, Fgfr4) were analyzed. CR increased HNF4α mRNA in livers of Sirt1-LKO mice, but did not alter HNF4α mRNA in livers of WT or Sirt1-TG mice. CR decreased LRH-1 mRNA in livers of Sirt1-TG mice, but did not alter LRH-1 mRNA in livers of Sirt1-LKO or WT mice. CR tended to increase PGC-1α mRNA in livers of all three genotypes of mice. FXR mRNA in livers of Sirt1-LKO mice was lower than that in WT mice under AL feeding. CR decreased FXR mRNA in livers of WT and Sirt1-TG mice, but did not alter FXR mRNA in livers of Sirt1-LKO mice. CR decreased the mRNAs of SHP and Fgfr4 in livers of Sirt1-TG mice, but did not alter them in livers of Sirt1-LKO or WT mice (Fig 5A). In ileum, CR did not alter FXR mRNA in any of the three genotypes of mice. CR tended to decrease SHP mRNA in ilea of WT mice, but did not alter SHP mRNA in ilea of Sirt1-LKO or Sirt1-TG mice. Fgf15 mRNA in ilea of Sirt1-LKO mice was lower than that in WT mice under AL feeding, and CR did not alter Fgf15 mRNA in ilea of any of the three genotypes of mice (Fig 5B).

### BA Transport in Liver and Ileum

**Fig 6:**
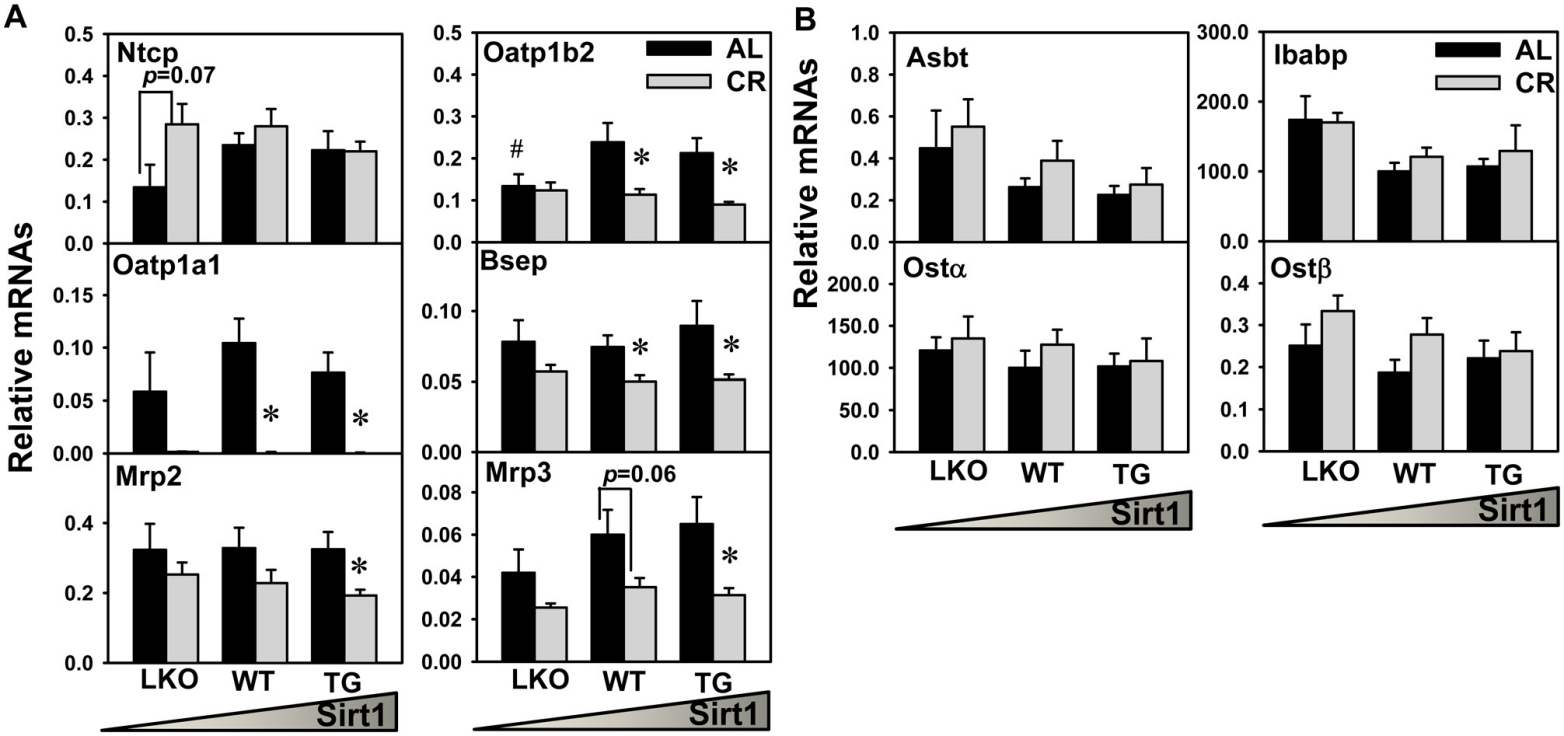
Expression of BA transporters in the enterohepatic circulation. The mRNAs of proteins involved in BA transport in liver (uptake: Ntcp, Oatp1b2, and Oatp1a1; efflux: Bsep, Mrp2, and Mrp3) (A) and ileum (uptake: Asbt; intracellular transport: Ibabp; efflux: Ostα, Ostβ) (B) were quantified. Data are presented as means ± SEM of 5 mice. The triangle represents increased expression of Sirt1 in liver. # represents differences compared to WT mice, by one-way ANOVA, followed by Duncan’s post-hoc test (*p* < 0.05). * represents differences compared to AL mice of the same genotype by Student's *t*-test (*p* < 0.05).

BA transporters in both liver and intestine promote BA enterohepatic circulation. BA reabsorption mainly occurs at the end of the small intestine (ileum), where BA transporters are mainly expressed. Thus, ileum tissue was used to determine the expression of BA transporters in intestine. As shown in Fig 6A, CR tended to increase Ntcp mRNA in livers of Sirt1-LKO mice, but did not alter Ntcp mRNA in livers of WT or Sirt1-TG mice. Oatp1b2 mRNA in livers of Sirt1-LKO mice was lower than that in WT mice under AL feeding. CR decreased Oatp1b2 mRNA in livers of WT (58%) and Sirt1-TG mice (58%), but did not alter Oatp1b2 mRNA in livers of Sirt1-LKO mice. CR decreased markedly Oatp1a1 mRNA in livers of WT (99%) and Sirt1-TG mice (99%), and tended to decrease Oatp1a1 mRNA in livers of Sirt1-LKO mice (98%). CR decreased Bsep mRNA in livers of WT and Sirt1-TG mice, but did not alter Bsep mRNA in livers of Sirt1-LKO mice. CR decreased Mrp2 mRNA in livers of Sirt1-TG mice, but did not alter Mrp2 mRNA in livers of Sirt1-LKO or WT mice. CR decreased Mrp3 mRNA in livers of Sirt1-TG mice, but did not alter Mrp3 mRNA in livers of Sirt1-LKO or WT mice. As shown in Fig 6B, CR did not alter in ileum the mRNAs of Asbt, Ibabp, Ostα, or Ostβ in any of the three genotypes of mice.

## Discussion

**Fig 7:**
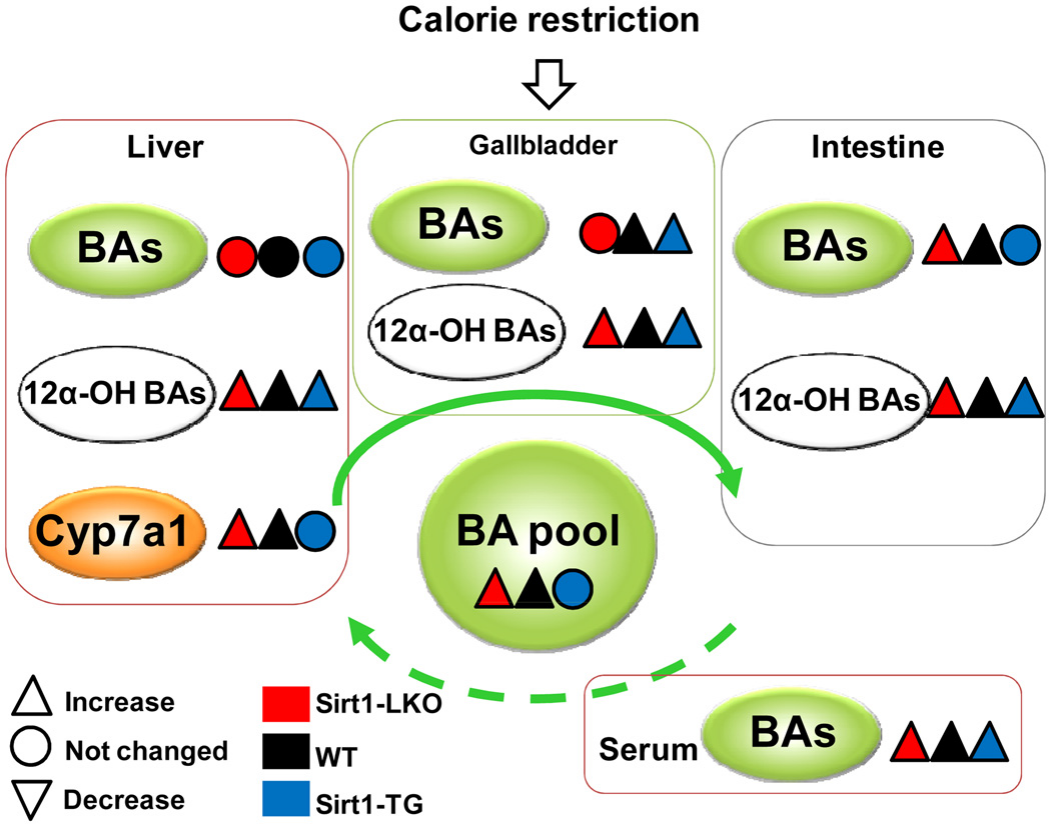
Summary of BA regulation during CR in various Sirt1 genetically-modified mice. In WT mice, CR increases the BA pool size and total BAs in serum, gallbladder, and small intestine. Additionally, the expression of Cyp7a1 also tends to be increased, suggesting more BA synthesis in liver. However, the CR-induced increase in BA pool size and Cyp7a1 expression is still observed with ablated expression of Sirt1 in liver, and completely suppressed with whole-body overexpression of Sirt1. Furthermore, CR-induced alterations in BA composition, especially the increase in the ratio of 12α- vs non-12α-hydroxylated BAs), remain similar in all three genotypes of mice.

The present study has utilized Sirt1 genetically-modified mice to investigate the role of Sirt1 in regulating BA homeostasis during short-term CR. To summarize the major findings (Fig 7), CR increases the BA pool size and total BAs in serum, gallbladder, and small intestine. CR also increases Cyp7a1 expression, suggesting increased BA synthesis in liver. However, the expression level of Sirt1 does not significantly affect the CR-induced alterations in BA pool size, BA profiles, and expression of BA-related genes.

CR increases BA pool size and the expression of the rate-limiting BA-synthetic enzyme Cyp7a1 in WT mice (Figs 2A and 4), which is consistent with our previous finding [33]. In agreement with the role of Cyp7a1 in determining the BA pool size [42], the CR-induced alterations in BA pool size and Cyp7a1 expression are consistent in Sirt1-LKO or Sirt1-TG mice. This suggests that increase in BA pool size during CR probably results from induced BA synthesis in liver.

The present study utilized mice of liver, rather than whole-body knockout of Sirt1, because most Sirt1 whole-body knockouts die in the perinatal period and the survivors have developmental defects [43]. CR increases the BA pool size in both WT and Sirt1-LKO mice (Fig 2A), which suggests that Sirt1 in liver does not mediate the BA changes during CR. Compared to WT mice, the ileal expression of Sirt1 is similar in LKO mice and higher (over 2-fold) in TG mice (Fig 1B), which is consistent with a previous report [24]. CR does not alter the ileal expression of Sirt1, regardless of Sirt1 genotype in mice (Fig 1B). This is different from a previous report that CR induced a 2-fold increase in Sirt1 protein in the intestine of rats [44]. This discrepancy is likely due to different animal model or CR feeding regime. Intestinal Sirt1 was recently shown to be required for ileal BA absorption and systemic BA homeostasis in mice [31]. The current finding that CR does not alter Sirt1 ileal expression suggests that Sirt1 in intestine does not mediate the BA changes during CR.

CR increases total BAs in serum, gallbladder, and small intestine, but BAs in liver and large intestine are not altered (Fig 2B and 2C). CR-induced Cyp7a1 expression and BA synthesis could lead to increased total BAs in serum, and the repressed hepatic expression of BA uptake transporter Oatp1b2 (Fig 6) may further contribute to the elevated serum BA levels. Gallbladder tissue with bile is used for BA extraction and quantification in the present study. CR is known to increase bile flow in rats [45], but the bile flow data are unavailable in the present study due to the technical challenge of performing bile duct cannulation on the small and lean CR mice. The small intestine contains the majority of BAs in the pool size, and the CR-induced BA changes in small intestine are consistent with the alteration in BA pool size in three genotypes of mice. BA reabsorption at the intestine might not be altered by CR, due to the constant expression of BA transporters (Asbt and Ostα/β) and intracellular transport protein (Ibabp) in ileum (Fig 6B). Collection of fecal samples for BA analysis in the future would provide information on whether the increase of BA pool size during CR could also be due to reduced fecal BA loss.

Individual BAs vary in physiochemical properties, potencies in promoting biliary cholesterol excretion and intestinal lipid absorption, as well as potencies in activating various receptors and signaling activities. Therefore, it is crucial to analyze not only the concentrations of total BAs but also the BA composition. It is known that 12α-hydroxylated BAs are implicated in the regulation of insulin signaling and lipid metabolism [36]. The present study shows that CR increases the proportion of CA and decreases the proportion of α/βMCA, and thus increases the ratio of 12α-hydroxylated BAs, regardless of Sirt1 genotype in mice (Fig 3C). Increased ratio of 12α-hydroxylated BAs is consistent with the improved insulin sensitivity and lipid metabolism during CR. Moreover, the CR-induced BA composition changes do not seem to be dependent on Sirt1.

Cyp8b1 is responsible for the synthesis of 12α-hydroxylated BAs in both the classic and acidic pathways [46]. Compared to WT mice, Sirt1-LKO mice have lower expression of Cyp8b1 in liver (Fig 4A), which correlates with the lower proportion of CA and lower ratio of 12α-hydroxylated BAs (Fig 3C). This is consistent with the understanding that Cyp8b1 determines the ratio of CA to CDCA in humans [47]. During CR, Cyp8b1 expression is decreased in WT and Sirt1-TG mice, but not altered in Sirt1-LKO mice (Fig 3), which is different from increased 12α-hydroxylated BAs in all three genotypes. Therefore, Cyp8b1 expression correlates with the ratio of 12α-hydroxylated BAs among three genotypes of Sirt1 mice, but does not seem to explain the CR-induced alterations in BA composition.

The present study further investigates the mechanism of CR-induced Cyp7a1 expression and BA increases. Nuclear receptors HNF4α and LRH-1 cooperate in the regulation of the promoter-mediated basal expression of Cyp7a1 [48]. FXR signaling in liver [49] and ileum [50] regulate the feed-back inhibition of Cyp7a1 transcription. Sirt1-LKO mice have lower expression of FXR in liver and Fgf15 in ileum than WT mice (Fig 5). This is consistent with a previous report of decreased FXR expression with Sirt1 deficiency in liver [32]. The CR-induced Cyp7a1 alterations in these genotypes of mice (Fig 4A) do not correlate with the negative regulators in FXR signaling (SHP in liver and Fgf15 in ileum) or the positive regulators (HNF4α and LRH-1 in liver) (Fig 5). In rodents, excessive cholesterol can stimulate BA synthesis by inducing Cyp7a1 expression through the cholesterol-sensor liver X receptor (LXR) [51]. The role of Sirt1 in regulation of LXR activation remains elusive due to contradictory findings [52,53]. During CR, cholesterol in liver is decreased [33] rather than increased, so it seems that LXR does not participate in Cyp7a1 induction during CR. Taken together, the CR-induced changes in Cyp7a1 expression cannot be totally explained by the conventional transcription factors discussed above.

PGC1α, a nutrient-sensitive metabolic regulator, is a co-activator for the promoter-mediated Cyp7a1 transcription [54]. Under nutrition deprivation such as CR, Sirt1 promotes fat mobilization and regulates hepatic glucose and lipid metabolism by activating PGC1α [55,56]. We and others have reported that PGC1α expression in liver was increased by CR in WT mice as well as Sirt1-LKO mice [22,23,33]. The present study shows that CR tends to increase PGC-1α expression in Sirt1-LKO, WT, and Sirt1-TG mice (Fig 5A). PGC1α expression seems to be induced by CR in a Sirt1-independent manner. This may provide explanation for the finding that Cyp7a1 expression can still be upregulated by CR in Sirt1-LKO mice (Fig 4A). Although Sirt1 can increase PGC1α activity through deacetylation, PGC1α can be regulated at transcriptional and post-translational levels by other factors, such as energy-sensor AMPK [57]. Therefore, CR-induced Cyp7a1 expression is likely due to the induced PGC1α expression.

In conclusion, CR increases Cyp7a1 expression and BA synthesis, and also increases the BA pool size and total BAs in serum, gallbladder, and small intestine. The repressed expression of BA uptake transporter Oatp1b2 in liver could also contribute to the elevated serum BAs. The CR-induced Cyp7a1 expression is likely due to PGC1α. The expression level of Sirt1 does not significantly affect CR-induced BA alterations.

## Abbreviations

AL: *ad libitum*
Asbt: apical sodium-dependent bile acid transporter
BA: bile acid
BAL: bile acid-CoA ligase
BAT: bile acid-CoA: amino acid *N*-acyltransferase; Bsep, bile salt export pump
CA: cholic acid
CDCA: chenodeoxycholic acid
CR: calorie restriction
Cyp: Cytochrome P450
DCA: deoxycholic acid
EHC: enterohepatic circulation
Fgf: fibroblast growth factor
FXR: farnesoid X receptor
GB: gallbladder
HDCA: hyodeoxycholic acid
HNF4α: hepatocyte nuclear factor 4 alpha
Ibabp: ileal bile acid binding protein
LCA: lithocholic acid
LI: large intestine; LRH-1, liver receptor homolog-1
LXR: liver X receptor
MCA: muricholic acid
MDCA: murideoxycholic acid
Ntcp: Na^+^/taurocholate cotransporting polypeptide
Oatp: organic anion transporting polypeptide
Ost: organic solute transporter
PGC-1α: peroxisome proliferator-activated receptor gamma coactivator 1-alpha
SHP: small heterodimer partner
SI: small intestine; Sirt, sirtuin
T-BA: taurine-conjugated bile acid
U-BA: unconjugated bile acid
UDCA: ursodeoxycholic acid
UPLC-MS/MS: ultra-performance liquid chromatography-tandem mass spectrometry
